# Supplementary material for: Shortened PGLYRP1 Peptides Regulate Antitumor Activity of Cytotoxic Lymphocytes via TREM-1 Receptor: From Biology to Bioinformatics
Source: Int J Mol Sci. 2025 Apr 25;26(9):4069. doi: 10.3390/ijms26094069 (PMC12071940; doi:10.3390/ijms26094069)
Supplement: Supplementary file 1 [file ijms-26-04069-s001.zip › ijms-3551137-supplementary.pdf]

Supplementary materials

Table S1. Intermolecular interaction observed between N15 and TREM-1. "Peptide" column stands for peptide residues involved in binding; "Protein" column stands for protein residues involved in binding; "Interaction type" column describes the observed type of intermolecular interaction; the "Occupancy" column describes the fraction of time though the whole trajectory the interaction is observed.

**Peptide**	**Protein**	**Interaction type**	**Occupancy**
ARG1	GLU30.A	HBDonor	0.5
		Cationic	0.5
		VdWContact	0.5
TYR2	LYS28.A	Hydrophobic	0.5
	ARG130.A	Hydrophobic	0.6
	VAL132.A	Hydrophobic	0.6
	PRO319.B	Hydrophobic	0.6
VAL4	LEU110.A	Hydrophobic	0.5
SER6	ILE58.A	HBDonor	0.8
		VdWContact	0.7
	ARG59.A	HBDonor	0.5
HIS7	ARG59.A	Hydrophobic	0.8
		PiCation	0.7
SER11	GLU106.A	VdWContact	0.7
CYS13	ILE85.A	Hydrophobic	0.9
	MET100.A	Hydrophobic	1
	ASP107.A	Hydrophobic	0.9

		VdWContact	0.9
	TYR111.A	Hydrophobic	0.6
		HBDonor	1
		VdWContact	0.8
ASN14	GLY83.A	VdWContact	0.5
	ARG84.A	Hydrophobic	0.5
		HBAcceptor	1
		VdWContact	1
THR15	LEU67.A	Hydrophobic	0.9
	VAL82.A	Hydrophobic	0.9
	ILE85.A	Hydrophobic	0.9

Table S2. Intermolecular interaction observed between 17.0 and TREM-1. “Peptide” column stands for peptide residues involved in binding; “Protein” column stands for protein residues involved in binding; “Interaction type” column describes the observed type of intermolecular interaction; the “Occupancy” column describes the fraction of time through the whole trajectory the interaction is observed.

ARG1	GLU33.B	HBDonor	0.7
		Cationic	0.7
		VdWContact	0.7
	VAL105.B	Hydrophobic	0.6
ASN2	GLU106.B	VdWContact	0.7
GLN4	ILE57.B	Hydrophobic	1.0
	GLU106.B	HBDonor	1.0
		VdWContact	1.0

	SER108.B	HBDonor	1.0
		VdWContact	0.9
HIS5	PRO119.A	Hydrophobic	0.8
	SER108.B	HBDonor	0.9
		VdWContact	0.8
	GLY109.B	VdWContact	0.7
	ARG130.B	Hydrophobic	0.7
	LEU131.B	HBDonor	0.7
		VdWContact	0.7
TYR6	GLN117.A	HBDonor	0.6
		VdWContact	0.6
	PRO119.A	Hydrophobic	1.0
		VdWContact	0.9
	ILE58.B	Hydrophobic	0.7
		VdWContact	0.5
	ASP60.B	Hydrophobic	0.9
	LEU110.B	Hydrophobic	0.6
MET8	TYR116.A	Hydrophobic	0.7
	PRO118.A	Hydrophobic	0.6

Table S3. Intermolecular interaction observed between N9 and TREM-1. “Peptide” column stands for peptide residues involved in binding; “Protein” column stands for protein residues involved in binding; “Interaction type” column describes the observed type of intermolecular interaction; the “Occupancy” column describes the fraction of time through the whole trajectory the interaction is observed.

Peptide	Protein	Interaction type	Occupancy
PRO1	ILE57.B	Hydrophobic	0.5
	GLU106.B	HBDonor	0.8
		Cationic	0.7
		VdWContact	0.9
	SER108.B	HBDonor	0.7
	SER108.B	VdWContact	0.7
ALA2	GLU106.B	HBDonor	0.6
		VdWContact	0.6
CYS4	ARG130.B	HBAcceptor	0.5
		VdWContact	0.6
	VAL132.B	Hydrophobic	0.5
GLN6	GLN117.A	Hydrophobic	0.8
		HBDonor	0.8
		VdWContact	0.8
	LEU110.B	Hydrophobic	0.8
GLN7	ARG72.A	HBAcceptor	0.5
ALA8	GLN52.A	HBAcceptor	1
		VdWContact	0.9
	TYR116.A	Hydrophobic	0.9
		VdWContact	0.5
	PRO118.A	Hydrophobic	0.8
ARG9	LYS47.A	HBDonor	0.8
		VdWContact	0.7
	SER50.A	HBDonor	0.7

		VdWContact	0.7
	SER51.A	HBDonor	0.7
		VdWContact	0.5
	ARG72.A	HBAcceptor	0.6
		Anionic	0.7
		VdWContact	0.7
	GLU121.A	HBDonor	0.8
		Cationic	0.8
		VdWContact	0.8

Supplemental Figure S1.

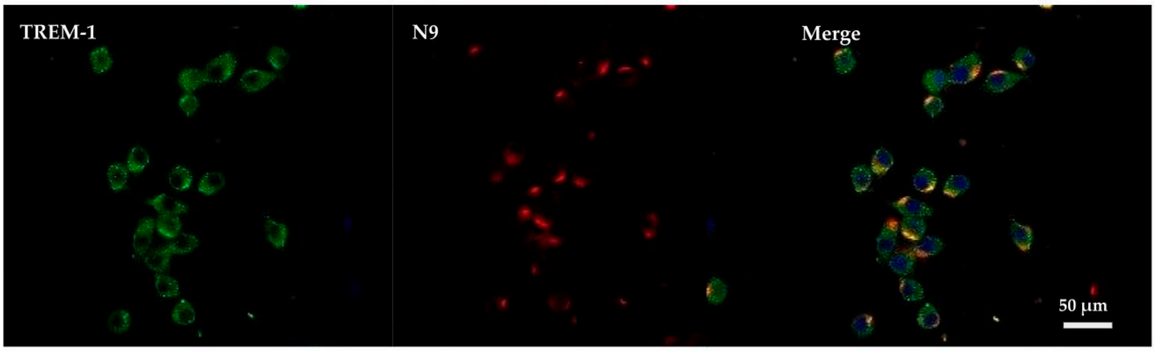

Typical confocal photos of N9 (red) and TREM-1(green) and layers superposition on the surface of RAW264.7 cells.

Supplemental Figure S2.

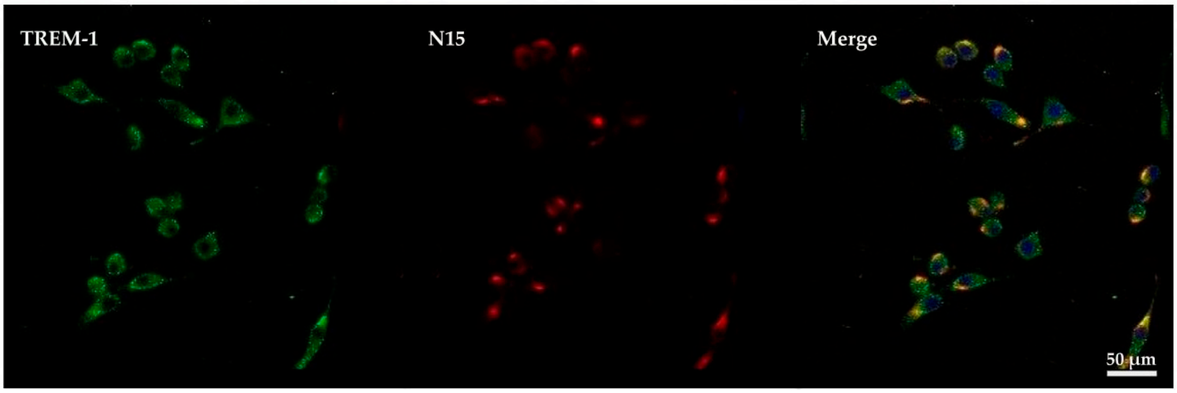

Typical confocal photos of N15 (red) and TREM-1(green) and layers superposition on the surface of RAW264.7 cells.

Supplemental Figure S3.

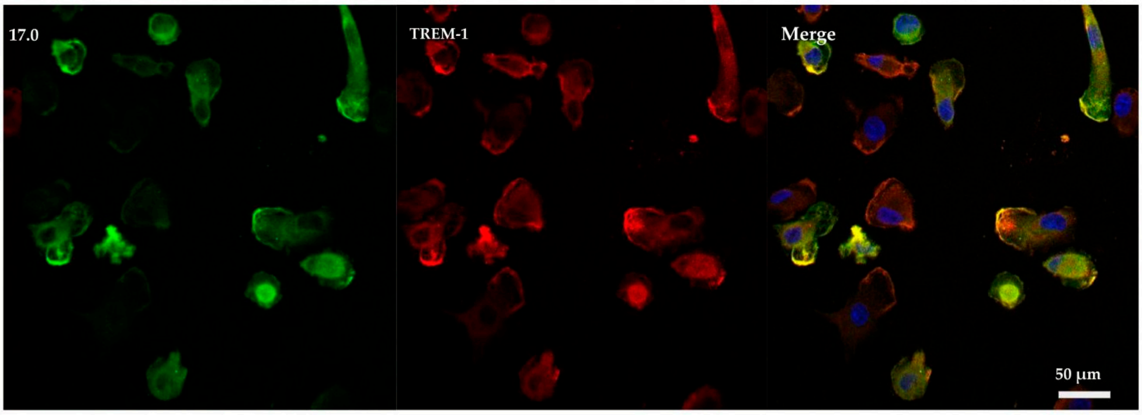

Typical confocal photos of 17.0(red) and TREM-1(green) and layers superposition on the surface of RAW264.7 cells.

Supplemental Figure S4.

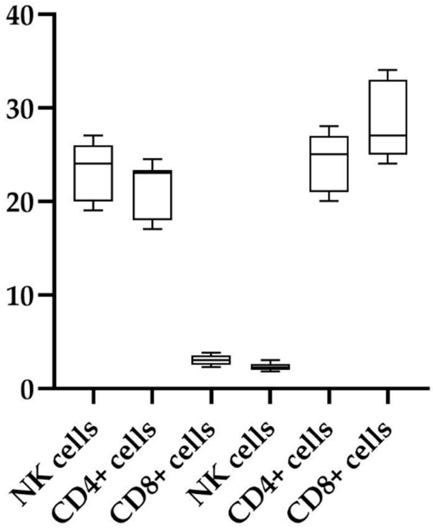

Cytotoxic activity of PBMC cells treated with the sum of N9+N15 peptides. Cell populations were isolated using magnetic separation.

Supplemental Figure S5.

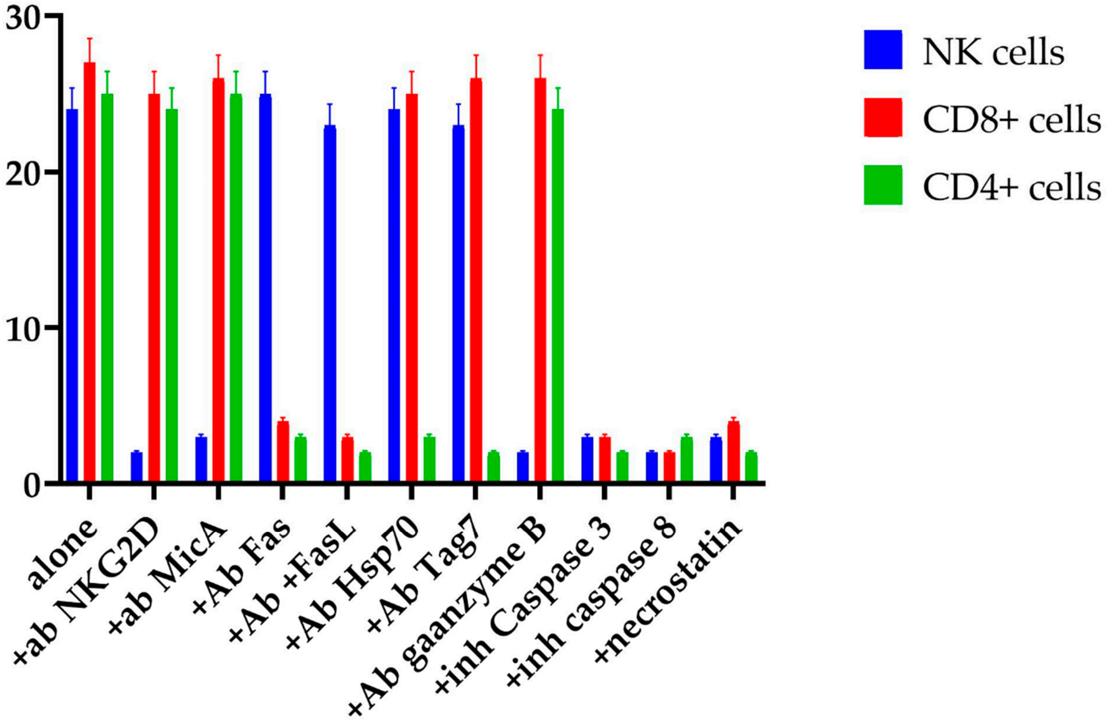

Cytotoxic activity of PBMC cells treated with the sum of N9+N15 peptides. Cell populations were isolated using magnetic separation on day 4 (NK cells) or day 6 (T lymphocytes) of incubation. Antibodies and inhibitors were added 1 hour prior cytotoxity detection.

Supplemental Figure S6.

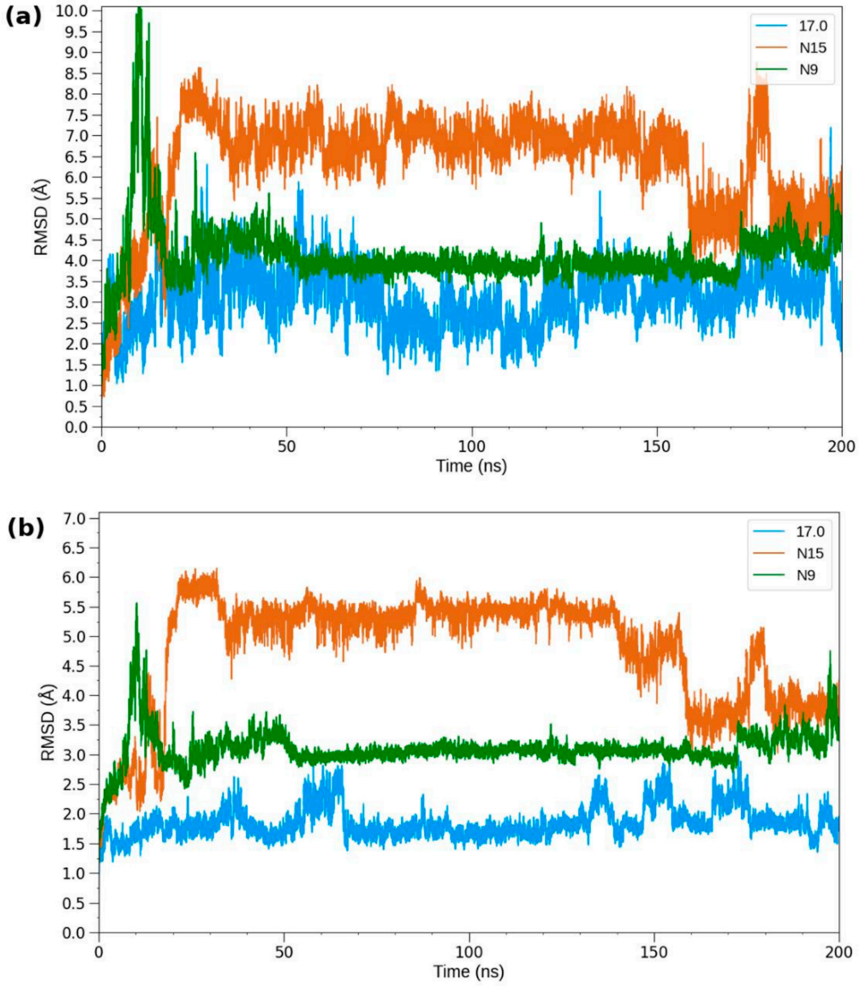

RMSD values of backbone atoms for peptides N9, N15, and 17.0 in complex with the dimer of water-soluble domain of the TREM-1 receptor. (b) RMSD values of atoms involved in peptide – TREM-1 interactions in peptides N9, N15, and 17.0.
